# Transcriptomic reprogramming of barley seminal roots by combined water deficit and salt stress

**DOI:** 10.1186/s12864-019-5634-0

**Published:** 2019-04-29

**Authors:** Alina Osthoff, Petra Donà dalle Rose, Jutta A. Baldauf, Hans-Peter Piepho, Frank Hochholdinger

**Affiliations:** 1 Institute for Crop Science and Resource Conservation, Crop Functional Genomics, University of Bonn, 53113 Bonn, Germany; 2 Institute for Crop Science, Biostatistics Unit, University of Hohenheim, 70599 Stuttgart, Germany

**Keywords:** Barley, Combined stress, RNA-Seq, Salt stress, Seminal roots, Transcriptome, Water deficit

## Abstract

**Background:**

Water deficit and soil salinity substantially influence plant growth and productivity. When occurring individually, plants often exhibit reduced growth resulting in yield losses. The simultaneous occurrence of these stresses enhances their negative effects. Unraveling the molecular mechanisms of combined abiotic stress responses is essential to secure crop productivity under unfavorable environmental conditions.

**Results:**

This study examines the effects of water deficit, salinity and a combination of both on growth and transcriptome plasticity of barley seminal roots by RNA-Seq. Exposure to water deficit and combined stress for more than 4 days significantly reduced total seminal root length. Transcriptome sequencing demonstrated that 60 to 80% of stress type-specific gene expression responses observed 6 h after treatment were also present after 24 h of stress application. However, after 24 h of stress application, hundreds of additional genes were stress-regulated compared to the short 6 h treatment. Combined salt and water deficit stress application results in a unique transcriptomic response that cannot be predicted from individual stress responses. Enrichment analyses of gene ontology terms revealed stress type-specific adjustments of gene expression. Further, global reprogramming mediated by transcription factors and consistent over-representation of basic helix-loop-helix (bHLH) transcription factors, heat shock factors (HSF) and ethylene response factors (ERF) was observed.

**Conclusion:**

This study reveals the complex transcriptomic responses regulating the perception and signaling of multiple abiotic stresses in barley.

**Electronic supplementary material:**

The online version of this article (10.1186/s12864-019-5634-0) contains supplementary material, which is available to authorized users.

## Background

Natural abiotic stresses such as water deficit and high soil salinity are major factors threatening global crop production [1, 2]. Exposure of plants to these osmotic stresses results in loss of turgor and as a consequence potential disruption of membranes and proteins accompanied by rising levels of reactive oxygen species (ROS) [3]. This, in turn, leads to growth inhibition and loss of yield [4]. While shoot growth is reduced under these stress conditions, roots continue to elongate at a slower rate to ensure survival by extracting water and nutrients from deeper soil layers [5]. Perpetuated root growth is mainly regulated by abscisic acid (ABA), which interacts with auxin, cytokinin, and ethylene in a hormonal network [6]. In addition to physiological alterations, the effects of either water deficit or salinity on gene expression patterns in roots have been studied. For instance, mature chickpea roots displayed several sets of differentially expressed genes in response to either water deficit or salinity at different developmental stages [7]. Microarray experiments in roots and leaves of three-week-old barley plants subjected to both stress conditions individually demonstrated, that the number and function of differentially expressed genes strongly depend on stress type and duration [8]. A study comparing gene expression levels in salt and osmotic-stressed barley leaves and roots came to the same conclusion [9]. While these studies surveyed the transcriptomic response to individual stress types, the simultaneous occurrence of several stress types under field conditions can lead to more severe responses [10]. Combinatorial abiotic stress application typically results in negative and in a few instances in positive physiological interactions between stress types [11]. For instance, a combination of salt and heat stress in Arabidopsis led to a negative effect by significantly reducing biomass and rosette diameter and lower survival rate that exceeded the decreases under single stress conditions [12]. Similarly, tobacco showed reduced respiration under water deficit, while heat shock and combined stress treatments enhanced this response [13]. In barley, plant growth and chlorophyll content reflecting the photosynthetic rate, water, and osmotic potential were reduced when subjected to either water stress, salinity or a combination of both. Yet, plants were more vulnerable to the combinatorial treatment of these stress factors [14]. In contrast, exposing tomato plants to combined heat and salinity had a positive effect leading to a significantly increased protection from the harmful effects of the individual application of salinity by accumulating trehalose and glycine betaine [15].

On the molecular level, it was demonstrated in early cDNA microarray studies in tobacco that the effects of combined water deficit and heat shock cannot be deduced by characterizing responses to single stress treatments [13]. Similarly, in Arabidopsis, the comparison of differentially regulated genes revealed a large overlap between heat and combined stress treatments but also a substantial treatment specificity [12]. In line with observations on the gene expression level, metabolic profiling of maize shoots and leaves treated by a combination of water deficit and salinity stress demonstrated that metabolic adjustments to combined stress were not additive when compared to single stress factors [16].

Barley is better adapted to abiotic stresses than other cereal species such as wheat or maize and can thus be grown in harsher environments [17]. This makes barley an ideal model plant to study abiotic stress adaptions. The usage of high molecular weight organic osmotica such as mannitol or PEG8000 (polyethylene glycol) to establish defined water potentials allows studying plant responses under controlled conditions. It was previously demonstrated that PEG8000 solution can be utilized to mimic water deficit [18–21]. Water deficit treatment of -0.8 MPa is in the mid-range of naturally occurring, plant-usable soil water potentials [22]. Similarly, NaCl concentrations of 150 mM in soil water is considered as moderate salinity and observed in many agricultural regions of the globe [23]. In the present study, we subjected 3-day-old barley seedlings to either PEG8000 solution with a water potential of -0.8 MPa to mimic water deficit, 150 mM NaCl to simulate salt stress or a combination of both. In these seedlings, we monitored root growth for eight consecutive days. Based on the results of these phenotyping experiments, we analyzed samples of seminal roots 6 h and 24 h after stress induction by RNA-Sequencing (RNA-Seq). The aim of this study was to explore the early transcriptomic reprogramming of barley seminal roots exposed to individual and combinatorial stresses at two time points. This study will provide candidate genes for further genetic analyses that might be helpful for marker-assisted barley breeding programs.

## Results

### Phenotypic response to abiotic stress treatments

**Fig. 1:**
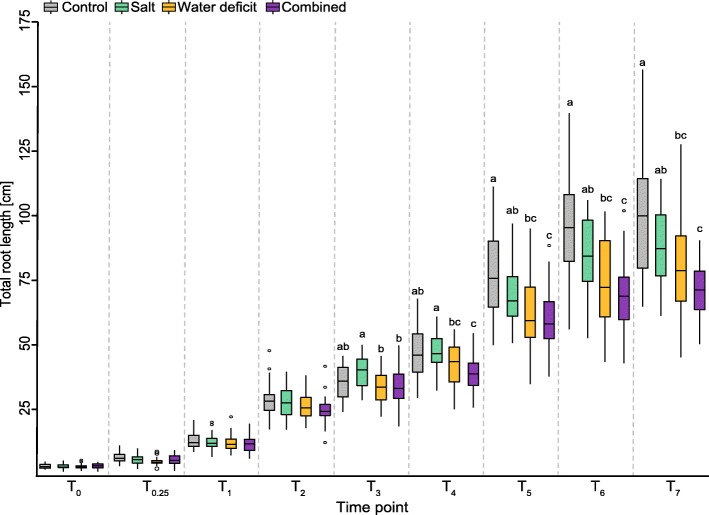
Comparison of total root length between control, salt, water deficit and combined stress from stress induction (T_0_) until 7 days of treatment (T_7_). Significant differences (α = 0.05) of means at each time point were calculated by ANOVA and indicated with small letters. Means not sharing any letter are significantly different

Seedlings of the barley spring cultivar Scarlett germinated for 2 days under control conditions were subjected to water deficit (PEG8000: -0.8 MPa), salt stress (NaCl: 150 mM) or a combination of both at T_0_ for 7 days (Fig. 1). To investigate the effect of the abiotic stress factors on seminal root development, total root length per treatment was determined relative to roots grown under control conditions (Fig. 1). By day four (T_4_), total seminal root length of seedlings subjected to combined stress treatment was significantly shorter than that of control plants (Fig. 1). By day five (T_5_), plants subjected to water deficit displayed also significantly reduced total root length relative to control plants. In contrast, although a substantial decrease in total root length was monitored in salt-stressed plants these differences were not statistically significant compared to control plants within 7 days of treatment.

### Mapping of RNA-sequencing reads to the barley reference genome

We monitored global changes in the seminal root transcriptomes of young barley seedlings subjected to water deficit, high salinity and a combination of both for 6 h and 24 h. These treatments correspond to time points T_0.25_ and T_1_ in Fig. 1. Hence, at both time points, no morphological differences between control plants and plants subjected to the three types of stress were detectable. Total RNA from four biological replicates per treatment-by-time point combination was extracted from seminal roots, converted into cDNA and subjected to RNA-Seq. The workflow of the RNA-Seq experiment and downstream analyses are summarized in Additional file 1: Figure S1. After quality trimming, between 67 and 76% of the obtained sequences per library mapped to the barley reference genome (Additional file 2: Table S1). After removal of stacked reads, i.e. reads that share identical 5′ coordinates, orientation and length, on average 60% of the remaining reads mapped successfully in pairs to the set of 39,734 high confidence gene models of barley version IBSC v2.0 [63].

### Transcriptomic relationships of RNA-Seq samples

**Fig. 2:**
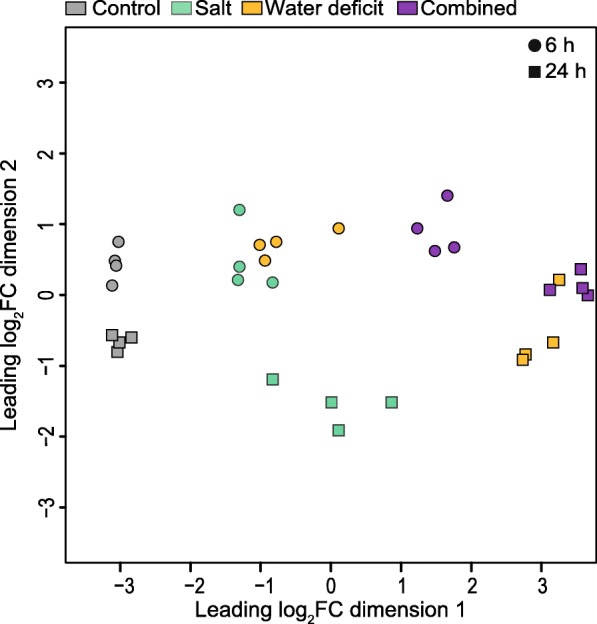
Multidimensional scaling plot of replicated RNA-Seq samples. Features represent libraries from control, water deficit, salt and combined stress treatment after 6 h and 24 h. Samples are arranged based on their calculated distances

Transcriptomic relationships between the type and duration of stress treatment were determined in a multidimensional scaling (MDS) plot (Fig. 2). Replicated samples of treatment-by-duration combinations clustered closely together. Moreover, samples subjected to short and long-term stress as well as distinct stress treatments are separable in the MDS plot, demonstrating that the observed transcriptomic divergence is driven by stress type and duration. For both stress durations, control and combined stress samples are positioned most distantly apart, while individually salt stressed and water stressed samples cluster between the control and combined treatment.

### Identification of differentially expressed genes

**Fig. 3:**
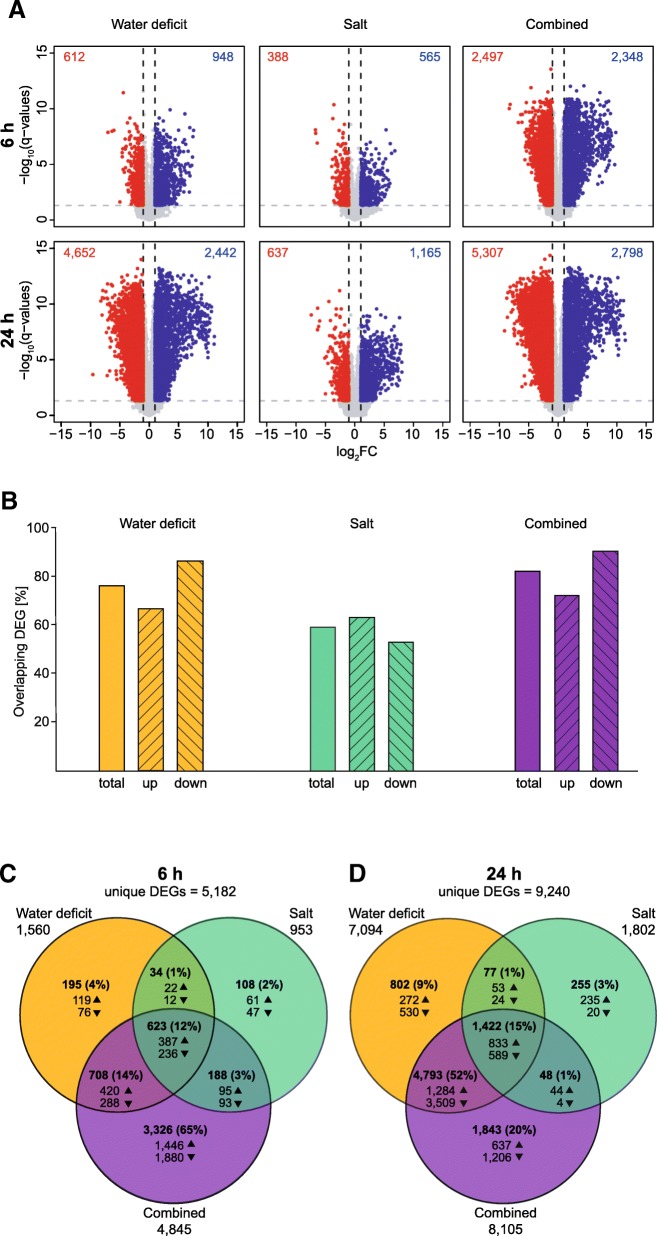
Overview of differentially expressed genes (DEGs) between control and stress-treated samples. **a** Volcano plots depict DEGs for each treatment-by-time combination. Up-regulated DEGs are indicated by blue dots, down-regulated DEGs are indicated by red dots. Total number of DEGs are shown in the upper left and right corner for significant up- and down-regulated DEGs. DEGs that do not exceed the threshold of |log_2_FC| >1 and FDR ≤1% are depicted in grey. **b** Overlap of DEGs at 6 h that are also differentially expressed at 24 h for each treatment in percent. Bars show overlaps of all DEGs, up-regulated and down-regulated DEGs separately. **c** Venn diagram showing the overlap between DEGs responsive to water deficit, salt and combined stress after 6 h of treatment. Arrows indicate number and direction of DEGs. **d** Venn diagram showing the overlap between DEGs after 24 h of treatment. Arrows indicate number of up- and down-regulated DEGs

Differentially expressed genes (DEGs) were computed in three pairwise contrasts between control and stress treatment samples for short- and long-term response. The number of DEGs (FDR ≤5% and |log_2_FC| >1) between control and stress treatment for the three treatment by two time point combinations are depicted as volcano plots (Fig. 3a). A comprehensive list of these DEGs is provided in Additional file 3: Table S2. The number of DEGs varied between treatment-by-time combinations. Under both, short and long-term stress exposure, the salt stress treatment resulted in the smallest number of DEGs (953 at 6 h and 1802 at 24 h). The most severe impact on gene expression was observed in the combined water deficit and salt treatment with 4845 DEGs at 6 h and 8105 DEGs after 24 h. After short-term treatment, the total number of genes differentially regulated in the combined treatment was substantially higher than the sum of genes differentially regulated by water deficit or salt stress alone. Furthermore, the direction of regulation depended on stress type and duration. Between 60% (salt treatment) and 80% (combined treatment) of genes that were differentially expressed after 6 h, were also responsive after 24 h of treatment (Fig. 3b). Among these, 70 to 75% of up-regulated DEGs and 55 to 95% of the down-regulated DEGs were conserved over time. Cross-comparison between the different gene sets after 6 h showed that the highest proportion of genes (65%) was unique to the combination treatment, while water deficit (4%) and salt (2%) treatment resulted in less uniquely expressed genes (Fig. 3c). This indicates that the combined treatment does not only result in the additive regulation of genes differentially expressed in the two single stress treatments. Instead, a substantial number of genes was only regulated by the combined stress but not by the individual stress factors. A set of 623 DEGs (12%) was responsive to all three treatments, pointing towards regulatory changes that were unaffected by stress type. Long-term stress response showed a strong overlap (52%) of genes responsive to water deficit and combined stress treatments, which is in line with the distribution of samples in the MDS plot in Fig. 2. Nevertheless, 20% of DEGs were specific for combined stress (Fig. 3d), while only 9 and 3% of DEGs were unique to water deficit and salt treatment, respectively.

### Assessment of stress-responsive pathways

Gene Ontology (GO) terms were assigned to DEGs to functionally characterize stress-responsive processes and functions. GO terms were analyzed for singular enrichment and obtained results were cross-compared with the SEACOMPARE tool. A full list of enriched GO terms in all treatment-by-time combinations is provided in Additional file 4: Table S3. In total, 63 GO terms responsive to short-term stress remained after filtering with REVIGO. Half of these terms were treatment-specific, while the other half was shared between two or more treatments (Table 1). The highest number of unique treatment-specific GO terms was observed for the combinatorial treatment. A substantial number of GO terms was commonly enriched between combined stress and each of the single stresses but not between water deficit and salinity. Shared terms of biological processes and molecular functions that were responsive to salt and combined treatment were commonly down-regulated. This included several catalytic activities such as ‘transferase activity’ (GO:0016740) but also metabolic processes like ‘phosphorus metabolic process’ (GO:0006793). Mutual DEGs between water deficit and combined stress were identified to be involved in oxidative and general stimulus responses. While ‘oxidoreductase activity’ (GO:0016491) and ‘oxidation-reduction process’ (GO:0055114) were down-regulated, responses to stimuli were up-regulated. The last set of treatment-independent terms showed up-regulation in all biological processes mainly involved in ‘transcription’ (GO:0006351), ‘regulation of gene expression’ (GO:0010468) and the corresponding functional term ‘transcription factor activity’ (GO:0003700). Genes involved in binding and hydrolase activity were down-regulated.

**Table 1:**
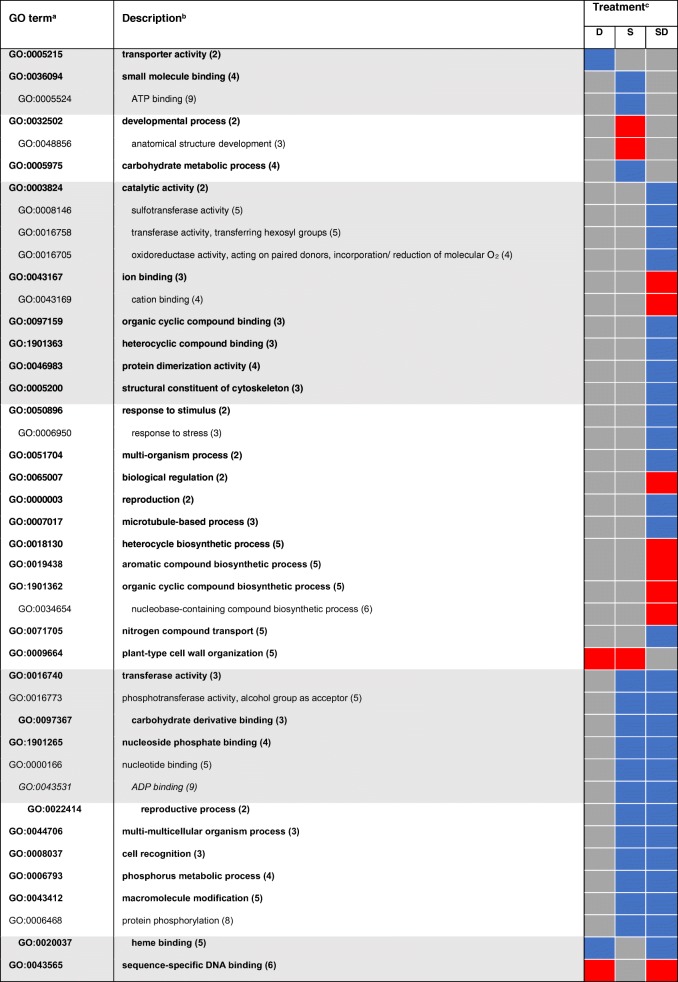
Enriched functional GO terms among DEGs responding to 6 h short-term treatment

^a^Only non-redundant terms (similarity ≤0.5) with FDR ≤5% are shown for identified molecular processes (white background) and molecular functions (grey background)

^b^Indented terms belong to the same cluster as the above listed higher-ranking term. Numbers in parentheses indicate the level of the GO term

^c^Treatments are water deficit (D), salt (S) and combined (SD). The direction of regulation is represented by blue (down-regulation), red (up-regulation), and grey (not significantly regulated)

GO terms assigned to DEGs after long-term exposure were mostly shared between two or more treatments (Table 2). A considerable number of commonly enriched terms was identified between water deficit and combined treatment, which is in support with the MDS plot (Fig. 2) and in line with the overlap of differentially expressed genes in Fig. 3d. Among functional terms, several transferase activities and metabolic processes were down-regulated, while ‘regulation of gene expression’ (GO:0010468) and ‘ion binding’ (GO:0043167) were up-regulated. Up-regulation of ‘developmental process’ (GO:0048856) and its child term was shared between water deficit and salt treatment, in contrast to salt and combined treatment, which showed no overlapping terms. Terms covered by ‘catalytic activity’ (GO:0003824) such as oxidoreductases, that were previously only enriched in short-term combined stress were enriched independent of stress type after long-term exposure. Furthermore, if terms were shared between two or more stress types, the direction of regulation was largely preserved. Up-regulation of the term ‘transcription factor activity’ (GO:0003700) was even conserved in all treatment-by-time combinations.

**Table 2:**
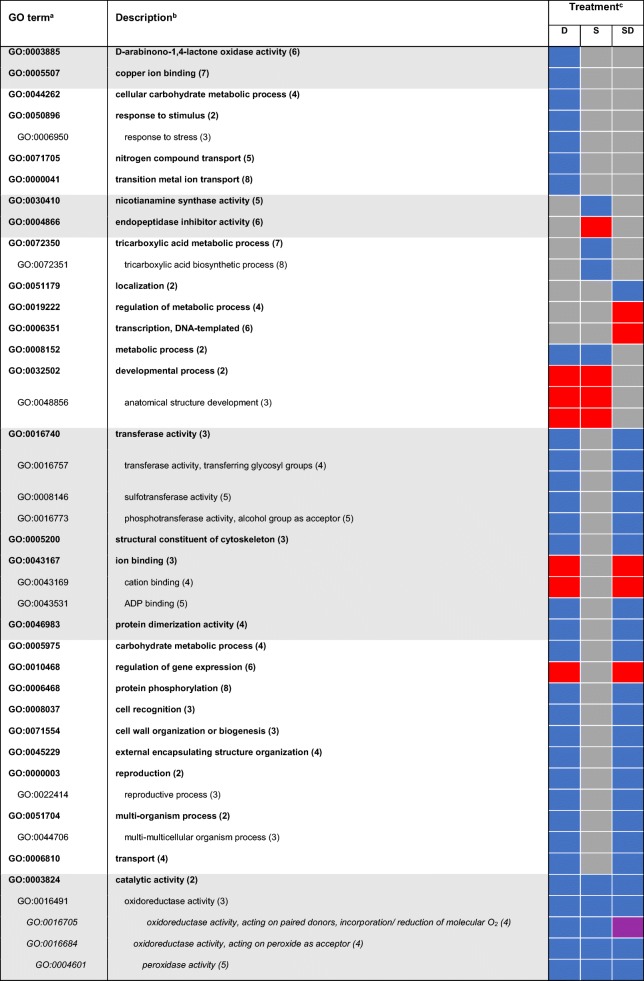
Enriched functional GO terms among DEGs responding to 24 h long-term treatment

^a^Only non-redundant terms (similarity ≤0.5) with FDR ≤5% are shown for identified molecular processes (white background) and molecular functions (grey background)

^b^Indented terms belong to the same cluster as the above listed higher-ranking term. Numbers in parentheses indicate the level of the GO term

^c^Treatments are water deficit (D), salt (S) and combined (SD). The direction of regulation is represented by blue (down-regulation), red (up-regulation), purple (up and down-regulation), and grey (not significantly regulated)

### Distribution of transcription factors in differentially expressed gene sets

**Fig. 4:**
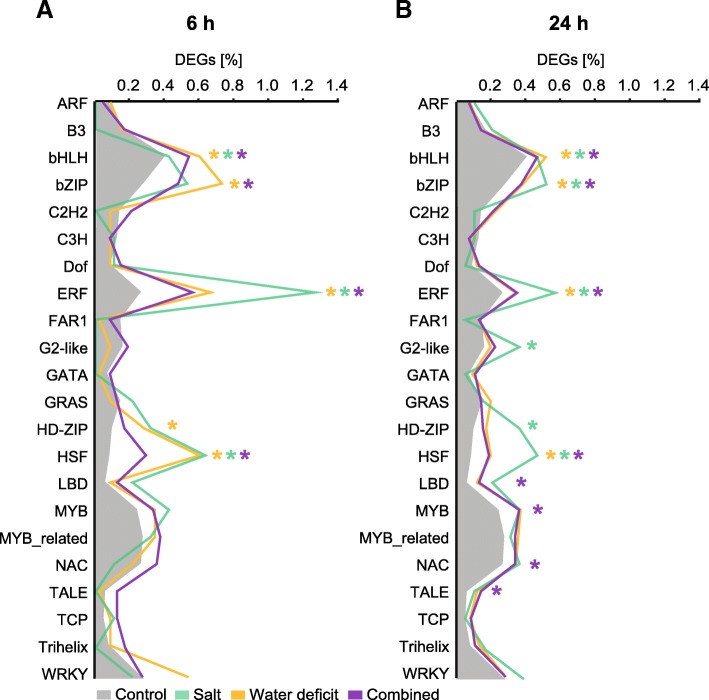
Prevalence of transcription factor (TF) families after salt, water deficit and combined stress for 6 h (**a**) and 24 h (**b**). Only families with ≥15 expressed members are shown. Grey background represents the family distribution among all expressed genes. Colored lines represent treatment-specific distributions of differentially expressed TFs as a percentage of all differentially expressed genes in the treatment. Significant deviations from the background distribution were calculated by Fisher‘s exact tests (α ≤0.05) and are indicated by asterisks

Transcription factors (TFs) within the RNA-Seq dataset were identified via the Plant Transcription Factor Database. In total, 924 of 2620 known barley TFs were expressed in the present dataset. The prevalence of these TFs in the 56 TF families was used as a reference distribution to identify deviations in the family distributions of the DEG datasets for each treatment-by-time combination (Fig. 4). Short-term 6 h stress treatment resulted in the enrichment of 12 treatment-by-TF family combinations (Fig. 4a) while 24 h stress led to the enrichment of 18 such combinations (Fig. 4b). At both stress treatment durations, 6 h and 24 h, bHLH, ERF, and HSF TF families were enriched at all three stress treatment combinations (Fig. 4a and b). In addition, the bZIP TF family was over-represented after water deficit and combined treatment at both time points (Fig. 4a and b). At 6 h, only the HD-ZiP TF family was enriched specifically upon water deficit treatment. At 24 h the bZIP, G2-like, HD-ZIP TF families were specifically enriched upon salt stress, while the LBD, MYB, NAC and TALE TF families were enriched upon combined salt and water deficit treatment. Remarkably, while several TF families were particularly enriched after 24 h after salt treatment, no water deficit-specific enrichment of TF families was observed after 24 h.

## Discussion

Under field conditions, crops are often subjected to simultaneous abiotic stresses such as water deficit, heat or high salinity [10, 11]. Understanding the molecular responses to these combined stresses that have detrimental effects on crop productivity is necessary for sustainable agriculture under changing global climatic conditions [11]. In the present study, we surveyed the individual and simultaneous effects of water deficit (PEG8000, -0.8 MPa) and high salinity (150 mM NaCl), on root development and the global transcriptome profiles of barley seminal roots.

Growth arrest as a response to salinity or water deficit in aboveground parts of plants is a common mechanism to conserve carbohydrates and thus maintain the energy supply. However, roots continue to elongate albeit at a lower rate to access water stored in deeper soil layers [24]. In the present study, we also observed a reduction in root growth rate. Roots of plants grown under combined stress and water deficit conditions continued to elongate but were significantly shorter in comparison to the roots of control plants after four or more days of treatment (Fig. 1). These phenotypic adjustments were also monitored in 12-day-old barley seminal roots, which were significantly shorter upon water deficit conditions of -0.8 MPa compared to control conditions [18]. In maize seedlings, phenotypic plasticity of primary roots in response to water deficit was even faster than in barley as demonstrated by a 30% reduced elongation within 24 h under the same -0.8 MPa water deficit regime as applied in the present study [20]. In contrast to water deficit, exposure to 150 mM NaCl solution did not affect root elongation in barley seedlings in the present study (Fig. 1). This supports the notion that salt tolerance of barley is linked to better root growth rates to provide an additional surface for sequestration of toxic ions that accumulate due to raising Na^+^ level within the plant [25].

To characterize the transcriptomic landscape of barley seminal roots and its adaptions in response to different abiotic stresses, seminal roots exposed to stress conditions for 6 h and 24 h were analyzed by RNA-Seq. Although significant developmental differences became only visible after 4 days in barley, previous studies showed, that transcriptomic adaptions are detectable already after a few hours of treatment and preceded the later observed phenotypic effects [8, 26, 27]. The number of identified DEGs, based on pairwise comparisons between control and stress samples, varied substantially between the duration of treatment with 5182 DEGs after 6 h (Fig. 2c) and 9240 DEGs after 24 h (Fig. 2d). This increase over time was for instance also reported in maize [20] and Arabidopsis [28]. Differences in the number of differentially regulated genes were also observed between different stress types. In line with their moderate impact on root elongation, salt treatment resulted in a considerably lower number of DEGs than water deficit at both time points (953 vs 1560 at 6 h and 1802 vs 7094 at 24 h; Fig. 3a). This remarkably low quantity of salt-responsive genes is in line with the previously demonstrated salt tolerance of barley [8]. Previous research suggested a possible link between the number of responsive genes and their association with the complexity and intensity of the imposed stress treatment. For instance, experiments in soybean exposed to different levels of water deficit showed that more severe stress treatment leads to an increased number of DEGs [29]. Furthermore, changes in stress complexity by applying multiple biotic and abiotic stresses to Arabidopsis were also positively correlated with the number of responsive genes [30]. In contrast to this, exposure of *Brachypodium distachyon* to triple stress (heat, water deficit, and salinity) did not increase the number of DEGs compared to double stress combinations [31]. The results obtained in this study support the notion that the duration of individual or combined stresses increases the number of differentially expressed genes (Fig. 3a). At the same time, genes regulated by these stresses at different time points displayed a substantial degree of conservation of 60 to 80% (Fig. 3b). Similar proportions of conservation of stress-responsive genes were also discovered in maize roots subjected to 6 h and 24 h of water deficit [20]. This finding supports the notion that certain molecular stress responses, which are already established after short-term exposure to stress, are still important after long-term exposure. Comparison of the DEG sets for each time point across treatments revealed that 65% of all short-term stress-responsive genes were unique to the combined stress treatment and as such not predictable by single-stress responses. This is consistent with findings in Arabidopsis ecotypes that also exhibited non-additive effects for plants subjected to combinatorial stress for 61% of the identified DEGs [32]. A similar pattern was observed in *Dianthus spiculifolius* subjected to cold and water stress, in which approximately half of the stress-responsive genes were unique to the combinatorial treatment after 24 h [33]. In the present study, only 20% of differentially expressed genes were unique to the combinatorial stress after 24 h, while a substantial overlap of regulated genes with water deficit regulated genes was observed. This notion is supported by recent studies in Arabidopsis suggesting that the response to one stress dominates the acclimation responses to a combination of stresses due to an extensive overlap between DEGs [12, 34].

Plant stress responses and adaptive processes to single and multiple stresses are orchestrated by a complex network of cross-talk between signaling pathways and sensors [10]. To gain further insight into biological processes and molecular functions that showed a stress-response, GO terms were assigned to the DEGs and analyzed for enrichment. Enriched GO terms observed in comparable studies in 12-day-old barley roots and young maize roots exposed to the same water deficit treatment are in accordance with the results of the present study. For instance, GO terms related to stress or stimulus responses and oxidoreductase activity were also highly over-represented in both experiments [18, 20, 26]. Moreover, exposure of Arabidopsis and chickpea plants to drought treatment resulted in an identical enrichment [7, 28]. Thus, indicating similar global patterns of stress response across species.

The abundance of enriched GO terms for both time points (Tables 1 and 2) further demonstrates the complexity of stress responses with the involvement of many different pathways. Water deficit and high salinity share a common osmotic component caused by a lowered water potential in the root vicinity [35] leading to identical responses and mutually enriched GO terms. The direction of regulation is highly conserved among these commonly enriched terms except for term GO:0009665 ‘plant-type cell wall organization’ which shows different directions between short and long-term stress and also between salt and other stress types. Within this GO term, expansins are highly represented. Continuous growth and development are based on constant loosening and remodeling of the cell wall that enables expansion [36]. Expansins play an important role in the regulation of these perpetual plasticity changes within cell walls [37]. Extensive studies in maize roots subjected to low water potentials linked enhanced gene expression of several expansins in the root growth zone to the maintenance of root growth under stress. Thus, alterations of root growth triggered by adjustment to water deficit are most likely due to the gene-specific regulation of expansin levels [38]. The regulation of expansin expression is in line with the previously described root growth adaptions. Under salt stress, expansins are up-regulated and thus, roots continue to elongate. This, in turn, can be linked to a better adaptation of barley to salt exposure [25]. In contrast, prolonged exposure to water deficit conditions and combined stress leads to down-regulation of expanins and to decreased root elongation as observed in later time points.

Another group of over-represented GO terms corresponded to antioxidant (GO:0016209) and oxidoreductase activity (GO:0016491) and their respective child terms. Genes associated with these functions are mainly involved in the scavenging of reactive oxygen species (ROS), known to harm plant cells subjected to different stresses [39]. Exposure to salt and water deficit resulted in a significant down-regulation of genes encoding oxidoreductases, glutathione reductases, and peroxidases. Down-regulation of involved enzymes was also observed in abiotically stressed Arabidopsis plants in which the direction of regulation strongly depended on the stress type underscoring the complexity of the ROS-induced network [40]. Several studies have examined expressional changes of genes involved in these processes when subjected to different stresses. In contrast to our study, 6 days of water deficit conditions in young barley roots resulted in the up-regulation of genes involved in oxidoreductase activities [18]. These findings suggest that genes involved in the ROS network are under developmental regulation and thus show different regulation over time.

Transcription factors (TFs) control the activity of downstream target genes. The GO term ‘transcription factor activity’ showed up-regulation in all investigated stress types and time points. In barley, 2620 TFs are classified in 56 families. Among those 924 TFs were active in seminal roots surveyed in the present study. A major proportion of these TFs are located within the bHLH, MYB-related and bZIP families [41].

Heat shock factors (HSFs) were significantly over-represented in all treatment-by-time combinations. HSFs control the expression of Heat-Shock-Proteins (HSPs) [42] that function as chaperones to protect proteins under heat stress [24, 43]. Nevertheless, it has been demonstrated that HSFs also play a role in general stress responses such as water deficit and combinations of non-thermal stresses [42, 44–46]. Experiments in barley showed that exposure to multiple abiotic stresses including water deficit and salinity resulted in the up-regulation of multiple HSFs. Of those, two candidates were subjected to qRT-PCR to validate the expressional changes [47]. We checked for these candidate genes in the present RNA-Seq dataset and found both genes HORVU4Hr1G090090 and HORVU4Hr1G090850 significantly up-regulated in all treatment-by-time combinations and with the highest fold change in the combined treatments. This alteration in modulation severity was also observed in experiments in Arabidopsis, that showed strong induction of HSFA7B by a combination of salt, osmotic and heat stress, while it was induced less severely under single heat stress [34]. The overexpression of *At*HSP17.6A in transgenic Arabidopsis plants led to enhanced osmotic stress tolerance [48]. Consequently, the expression of HSFs correlates with osmotic stress tolerance. Therefore, modulating the expression of HSF and HSP encoding genes might be an effective strategy for breeding plants with enhanced tolerance to abiotic stress.

Another over-represented family at all treatment-by-time combinations were ERF TFs. ERFs are involved in many developmental and physiological processes [49] but also act in response to wounding [50] and in abiotic stress response. Like HSF they have the potential to improve crop tolerance to abiotic stresses as demonstrated by transgenic plants overexpressing certain ERFs that are more resistant to salinity, cold and water stress [51, 52]. In the present study, genes identified as ERF were both up and down-regulated. While a swift induction of stress accelerates ethylene production, a moderate change results in inhibition of ethylene biosynthesis which in turn leads to different regulatory directions in gene expression [29]. Exposure of rice to high salinity and water deficit leads to the induction of two ERFs known as DREB1A and DREB2A [53]. In the present study, the barley homolog to DREB2A HORVU1Hr1G060490 was only slightly induced in long-term water deficit and combined stress. The closest barley homolog for DREB1A HORVU5Hr1G080450 was significantly down-regulated in response to both water deficit treatments but not by any other treatment-by-time combination.

The phytohormone ABA mediates gene expression by induction of ABA-dependent TFs from the bZIP and bHLH families. Both have regulatory functions in numerous developmental and physiological processes, including stress response [54, 55]. Enrichment of bHLH among differentially expressed genes was observed in all treatments and durations, while the bZIP family was enriched in all conditions except the salt treatment at 6 h. It was previously demonstrated in Arabidopsis that the large family of bHLH TFs contains members that regulate cell elongation and thus have a direct effect on the root development [56]. The function of these bHLH genes in barley needs to be elucidated in future genetic analyses.

## Conclusions

This data provides a starting point to understand the complex molecular mechanisms involved in the perception and signaling of multiple abiotic stresses in barley. Moreover, candidate genes identified here are a resource for further detailed genetic studies. Understanding the molecular networks underlying the signaling of combinatorial stresses will also be helpful for the identification of possible breeding targets for improved barley stress tolerance.

## Material and methods

### Plant material, growth conditions, and treatment

For phenotyping and transcriptome experiments, seeds of the German spring barley cultivar Scarlett were stratified in Petri dishes on soaked filter paper at 4 °C for 3 days to synchronize germination. Subsequently, seeds were transferred to germination paper (Anchor Paper Co, Saint Paul, USA) and grown in half-strength Hoagland solution [57] in growth cabinets (Conviron, Winnipeg, Manitoba, Canada) at 16 °C at night (8 h) and 20 °C at day (16 h). After 2 days of growth under control conditions, Hoagland solution was replaced and complemented with either PEG8000 solution (-0.8 MPa) to simulate water deficit, NaCl solution (150 mM) to induce salt stress or a combination of both.

### Phenotypic evaluation of seedlings

To facilitate image-based phenotyping, stratified seedlings were grown in germination pouches in custom-built boxes that were manufactured in-house for this purpose. Each box consists of 25 slots to fit growth pouches at an angle of 15° to ensure root growth along the germination paper and not the pouch foil. In each pouch, one seedling was grown. This experimental system enables easy handling without disturbing the roots when documenting them. The replicates for each treatment (control, water deficit, high salinity and combined) were allocated to the boxes in a randomized block design. In total, 25 replicates per treatment were measured in four boxes (blocks). To avoid exposure of the roots to light, the boxes were closed with lids until the initiation of shoot growth. Then, the top of the boxes was covered with aluminum foil sparing the emerging shoots. Seedlings were imaged prior to stress induction and after stress induction every 24 h for eight consecutive days. Total root length was measured with WinRHIZO Pro (Version 2009b, Regent Instruments, Canada) based on pixel color classifications. Obtained values were log_2_-transformed to meet the assumptions for an ANOVA. The block model included boxes as blocks (B) and pouches as plots (P) (B/P = B + B*P) with B*P as the residual error term. The following treatment model was applied: DxS = D + S + D*S with D = water deficit (control vs treated) and S = salt (control vs treated). The R package *ggpubr* (R Version 3.4.0, [58], ggpubr_0.1.6, [59]) was used for visual representation of the data. Statistical evaluation was performed with the packages *car* (car_2.1–6, [60]) for ANOVA and *agricolae* (agricolae_1.2–8, [61]) for Tukey’s tests.

### RNA isolation, cDNA library construction and RNA-Sequencing

For RNA extraction from seminal roots, ten seeds were grown in paper rolls as previously described [62]. Samples were harvested after 6 h and 24 h of treatment, immediately frozen in liquid nitrogen and stored at -80 °C until RNA extraction. For each replicate, ten roots were pooled. Total RNA was extracted with the RNeasy Mini Kit (Qiagen, Venlo, The Netherlands), according to the manufacturer’s instructions. RNA quality was checked with a Bioanalyzer (Agilent RNA 6000 Nano Chip, Agilent Technologies, Santa Clara, CA, USA). RIN (RNA integrity number) values ≥7.5 were obtained for all collected samples indicating their high quality and integrity. cDNA libraries for transcriptome sequencing were constructed according to the TruSeq RNA Sample Preparation protocol (Illumina, San Diego CA, USA). Library indexing, cluster preparation, and paired-end sequencing were performed according to the manufacturer’s instructions (Illumina). In total, 32 libraries with four biological replicates per treatment were sequenced on an Illumina HiSeq 4000 sequencer resulting in 100 bp paired-end reads.

### Processing of raw sequencing data

RNA-Seq reads were processed with CLC Genomics Workbench (Version 10.0.1; https://www.qiagenbioinformatics.com/products/clc-genomics-workbench/). The raw sequencing data were deposited in NCBI’s sequencing read archive under accession number SRP133479 (https://www.ncbi.nlm.nih.gov/sra/SRP133479). Low-quality reads and adapter sequences were removed from the dataset by trimming. Only reads with a length of ≥40 bp were retained for further analyses. The remaining reads were mapped to the barley reference genome of the genotype morex [63], ftp://ftp.ensemblgenomes.org/pub/plants/release-36/fasta/hordeum_vulgare/dna/; Hv_IBSC_PGSB_v2) allowing large gaps of up to 50 kb to span introns. Reads were mapped successfully when they matched uniquely with ≥80% of their length and ≥ 90% identity to the reference sequences. By mapping RNA-seq reads of the barley genotype Scarlett to the reference genotype Morex we introduce a mapping bias which is reflected by the average mapping rates of 72% in Additional file 3: Table S2. By this approach, we toss out many true positives (Additional file 2: Table S1). However, without a Scarlett reference genome at hand, we cannot decide which of these unmapped reads would map to unique and which would map to multiple positions in the Scarlett genome. Therefore, de novo assembly of unmapped reads could introduce substantial false positive rates that might affect gene expression patterns. We, therefore, decided to exclude reads that do not map to the Morex reference genome from further analyses. Stacked reads, i.e. read pairs that have identical 5′ coordinates, orientation and length, were removed from the dataset. Subsequently, the remaining reads were mapped to the set of high-confidence gene models [63], ftp://ftp.ensemblgenomes.org/pub/plants/release-36/gff3/hordeum_vulgare/; Hv_IBSC_PGS_v2.36). Only reads that matched with ≥90% of their sequence length and ≥ 90% identity to the longest transcripts of the high confidence gene models were considered as mapped. Multi-mapping reads that mapped to more than one position were excluded from subsequent analyses.

### Multidimensional scaling analysis

To assess the quality of the data, samples were clustered in a multidimensional scaling plot (MDS plot) using the plotMDS function implemented in the Bioconductor package *limma* in R (R Version 3.4.0, limma_3.32.2, [64]). Resulting distances between paired samples were displayed as the leading log_2_-fold change, which is defined as the estimated root-mean-square deviation for the top 500 genes with the largest standard deviation among all samples. This analysis provided a visual representation of sample relationships by spatial arrangement.

### Statistical assessment of differential gene expression

To meet the assumptions of a linear model, the obtained read counts were normalized by sequencing depth and log_2_-transformed. The mean-variance relationship was estimated and used to assign precision weights to each observation to adjust for heteroscedasticity [65]. A linear model was fitted to assess differences in gene expression between control and stress treatments at 6 h and 24 h. The model included a fixed effect for treatment and time and the interaction of both terms. To estimate the variability over all genes and to shrink the variances towards a common value, an empirical Bayes approach was applied [66]. The contrasts.fit function of the R package *limma* was used to compute pairwise comparisons between each stress and control treatment at 6 h and 24 h and between short and long-term stress induction for each treatment. To correct the calculated *p-*values of the performed pairwise *t*-tests for multiplicity, the false discovery rate (FDR) was adjusted to ≤5% [67].

### Gene ontology (GO) and transcription factor analyses

To gain better insight into stress-responsive pathways, GO categories were assigned to differentially expressed genes with the web-based agriGO v2.0 software [68]. Singular enrichment analysis identified over-represented categories by comparing GO terms of up and down-regulated differentially expressed genes separately to the set of all expressed genes based on Fisher’s exact test. To correct for multiple testing, the resulting *p*-values were adjusted by controlling the FDR ≤5% [67]. The obtained results were combined and cross-compared with the SEACOMPARE tool implemented in the agriGO v2.0 software [68]. REVIGO [69] was used to filter redundant GO terms based on their similarity. Only terms with a similarity of ≤0.5 were kept.

Transcription factors were identified by sequence similarity searches of proteins from IBSC (The International Barley Sequencing Consortium) v1.0 annotation [70] deposited in the Plant Transcription Factor Database v4.0 [41] versus the barley gene annotation IBSC v2.36 [63] via blastp (https://blast.ncbi.nlm.nih.gov/Blast.cgi?PAGE=Proteins). All expressed transcription factors within the RNA-Seq dataset were separated for short and long-term responsive genes and assigned to 56 families. The same classification was performed for transcription factors identified as differentially expressed in each treatment-by-time combination. Significant shifts between the expected background distribution of all expressed transcription factors and the observed distribution of differentially expressed transcription factors were determined by Fisher’s exact test (α ≤0.05) for 6 h and 24 h separately.

## Additional files

Additional file 1: Overview of the experimental workflow of the RNA-Seq experiment. (PDF 130 kb)

Additional file 2: Overview of RNA-Seq output and mapping results. (XLSX 16 kb)

Additional file 3: Comprehensive list of the differential expression analysis. (XLSX 3317 kb)

Additional file 4: Overview of all identified over-represented Gene Ontology (GO) terms. (XLSX 28 kb)

## Abbreviations

DEGs: Differentially expressed genes
GO: Gene ontology
TFs: Transcription factors
