# Supplementary figures and images for: Transcriptomic reprogramming of barley seminal roots by combined water deficit and salt stress
Source: BMC Genomics. 2019 Apr 29;20:325. doi: 10.1186/s12864-019-5634-0 (PMC6489292; doi:10.1186/s12864-019-5634-0)

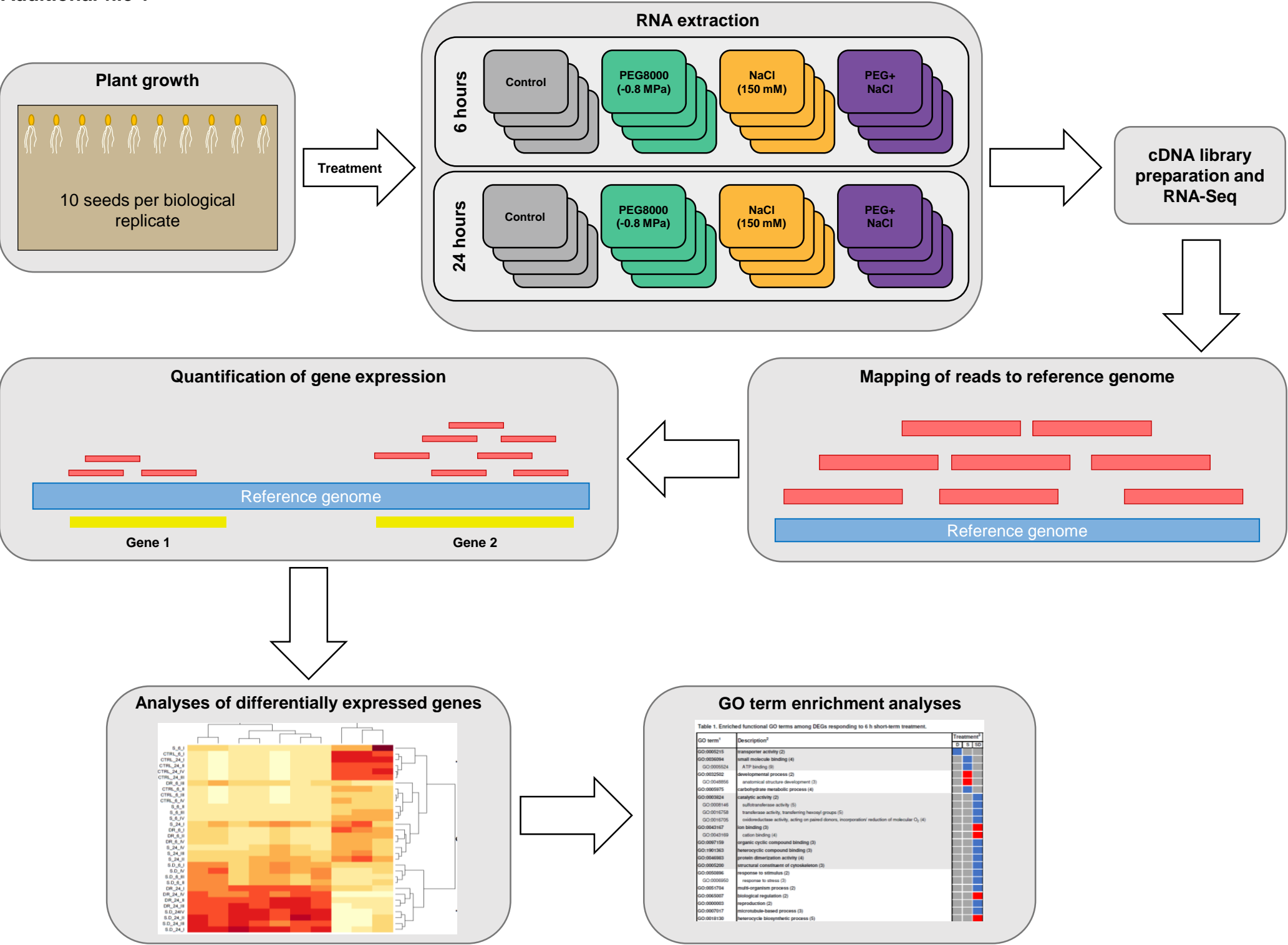

Supplement: Supplementary file 1 — Overview of the experimental workflow of the RNA-Seq experiment. (PDF 130 kb) [file 12864_2019_5634_MOESM1_ESM.pdf]
